# Portland Cement: An Overview as a Root Repair Material

**DOI:** 10.1155/2022/3314912

**Published:** 2022-01-06

**Authors:** Shahriar Shahi, Elaheh Fakhri, Hamidreza Yavari, Solmaz Maleki Dizaj, Sara Salatin, Khadijeh Khezri

**Affiliations:** ^1^Dental and Periodontal Research Center, Tabriz University of Medical Sciences, Tabriz, Iran; ^2^Department of Endodontics, Faculty of Dentistry, Tabriz University of Medical Sciences, Tabriz, Iran; ^3^Deputy of Food and Drug Administration, Urmia University of Medical Sciences, Urmia, Iran

## Abstract

Portland cement (PC) is used in challenging endodontic situations in which preserving the health and functionality of pulp tissue is of considerable importance. PC forms the main component of mineral trioxide aggregate (MTA) and demonstrates similar desirable properties as an orthograde or retrograde filling material. PC is able to protect pulp against bacterial infiltration, induce reparative dentinogenesis, and form dentin bridge during the pulp healing process. The biocompatibility, bioactivity, and physical properties of PC have been investigated *in vitro* and in animal models, as well as in some limited clinical trials. This paper reviews Portland cement's structure and its characteristics and reaction in various environments and eventually accentuates the present concerns with this material. This bioactive endodontic cement has shown promising success rates compared to MTA; however, considerable modifications are required in order to improve its characteristics and expand its application scope as a root repair material. Hence, the extensive chemical modifications incorporated into PC composition to facilitate preparation and handling procedures are discussed. It is still important to further address the applicability, reliability, and cost-effectiveness of PC before transferring into day-to-day clinical practice.

## 1. Introduction

Calcium silicate endodontic cements have indicated favorable clinical outcomes when used as orthograde or retrograde filling materials for vital pulp therapy and root obturation or to seal the root perforation [1]. Recently, the proposed management strategies for deep caries treatment recommend avoiding pulp exposure and highlight minimally invasive procedures. The challenge for the modern approaches in endodontic therapy is to promote remineralization of carious dentin and maintain pulp vitality. Various bioinductive materials have been derived from calcium silicate, the composition of which has been widely modified over the years leading to innovative clinical applications. In literature, these materials are referred to as “bioactive endodontic cements” due to their capacity to interact with living tissues and form an apatite-like layer on their surface when exposed to physiological fluids [2]. The most common cement with general use is Portland cement (PC), and it is a principal ingredient of concrete, stucco, plaster, mortar, and grout and consists predominantly of calcium, silicon, and aluminum oxides [3]. PC is made by heating limestone up to 1400°C with addition of clay and grinding the obtained product which is called clinker, with gypsum (CaSO_4_·4H_2_O) [4]. The main compounds of Portland cement are alite (tricalcium silicate (Ca_3_SiO_5_)), belite (dicalcium silicate (Ca_2_SiO_4_)), aluminate (tricalcium aluminate (Ca_3_Al_2_O_6_)), and ferrite (tetracalcium aluminoferrite (Ca_2_AlFeO_5_)) [2]. Based on different proportions of four major compounds, Portland cement is classified into five types: cement type *Ι* has high tricalcium silicate content with general uses while type *ΙΙ* has low tricalcium aluminate (<8%) and type *ΙΙΙ* has slightly more tricalcium silicate with finer particles and high early strength. Types *Ι*V and V possess lower tricalcium aluminate amounts [5, 6].

Alite is the major composition of Portland cement and is responsible for its setting and early strength. It reacts with water and forms seven polymorphic crystalline phases depending on the temperature and impurities [7]. Belite roughly reacts with water and forms calcium silicate hydrates (C-S-H) and portlandite (Ca(OH)_2_) and is mostly responsible for long-term strength development [8]. Addition of alumina during the process of making cement reduces the temperature and leads to the formation of aluminate in the resultant cement. Aluminate accelerates the setting procedure undesirably which is controllable by adding retardants [4]. Moreover, PC contains low levels of heavy metals (5 to 100 ppm) including arsenic, chromium, and lead which are added to the cement during the production process [9].

PC has been introduced to dentistry over one hundred years ago. However, its potential clinical applications became evident later. Commonly in dentistry applications, PC refers to cement variety type I [2]. The beneficial properties of PC including antibacterial activity [10], biocompatibility [11], bioinductivity [12], noncytotoxicity [13], good sealability, acceptable setting time, and physical and mechanical characteristics [5] are the rationale behind its widespread applications including the repair of root perforation and resorption, pulpotomy, and vital pulp therapy. Despite extensive experimental and animal studies on this cement, there are limited clinical trials. Thus, PC is now under investigation for various properties promoting its usage in clinical dentistry as an alternative to MTA [5]. New Portland cement-based endodontic cements such as TheraCal with additional components, predominantly radiopacifiers and resin, have been introduced and attracted attention for superior biological and mechanical properties.

This paper includes Portland cement publications in the dental field and related material studies from peer-reviewed journals published in English and discusses its chemophysical, mechanical, and biological characteristics as well as miscellaneous modifications incorporated into cement structure to facilitate the preparation and handling procedures.

## 2. Comparison of Portland Cement and MTA Compositions

**Figure 1:**
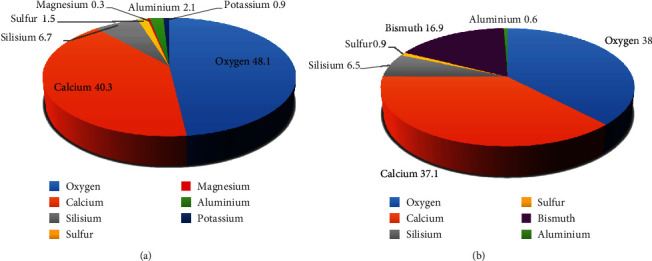
The main components of PC (a) and ProRoot MTA (b) according to EDX analysis.

MTA is a biocompatible material approved by the US Federal Drug Administration (FDA) with a wide range of applications in restorative dentistry and endodontics [14]. Although PC and MTA are almost identical in chemical composition and macro- and microscopic features, studies have reported differences in their various physical and biological properties. According to X-ray diffraction (XRD) analysis, MTA contains a high percentage of alite and belite; however, low levels of aluminate are detectable in MTA compared to PC [15]. ProRoot MTA contains 50-70% alite and 15-30% belite, which are so close to the weight percent of them in PC, but it has half the amount of gypsum of PC [16, 17]. To be specific, ProRoot MTA is composed of 19.8% bismuth oxide, 51.9% tricalcium silicate, 23.2% dicalcium silicate, which are the main components of Portland cement, 3.8% calcium dialuminate, and 1.3% calcium sulfate dehydrated [17, 18]. In other words, the essential difference between MTA and PC is the presence of bismuth oxide in MTA structure as a radiopacifier [16]. The main components of PC and ProRoot MTA according to energy-dispersive X-ray (EDX) analysis are presented in Figure 1 [16].

Overall, the similarity of ProRoot MTA and MTA Angelus to PC has been widely investigated [15, 19–24]. Funteas et al. found no difference in the amounts of 14 elements between ProRoot MTA and PC except for bismuth. Their study showed trace amounts of arsenic, barium, chromium, copper, iron, manganese, nickel, vanadium, and titanium in both materials [20]. Furthermore, low levels of zirconium have been reported in MTA composition [25]. According to EDX and X-ray photoelectron spectroscopy (XPS) analysis, MTA contains smaller quantities of aluminum, iron, sulfur, magnesium, and heavy metals such as copper, manganese, and strontium implying that lesser allergic and inflammatory reactions could possibly be diagnosed [16, 26, 27].

Scanning electron microscopy (SEM) showed that MTA is composed of homogeneous small particles with some larger ones dispersed in it, while PC has larger particles [16, 25, 28]. Assessment of particle size distribution of ProRoot MTA and MTA Angelus also has confirmed these results [29]. Cumulative percentage of particles with a size between 6 and 10 *μ*m is almost 70% for Portland cement. No noticeable difference has been reported between gray ProRoot MTA and PC in particle circularity and size distribution [30].

## 3. Chemical Properties

### 3.1. Setting Process

**Figure 2:**
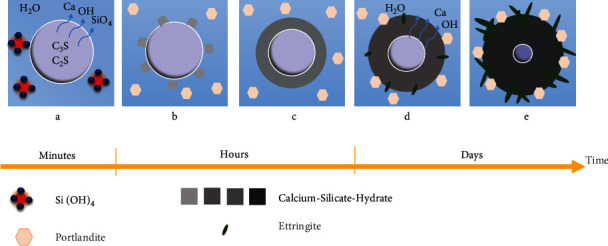
The hydration process of Portland cement.

Portland cement is hydraulic cement, which means it sets by mixing the powder with distilled water (Figure 2). Portland cement setting process involves progressive hydration of the orthosilicate ions (SiO^4-^) of tricalcium and dicalcium silicate and mainly results in calcium-silicate-hydrate (C-S-H) gel and rhombohedral crystals of portlandite (crystalline calcium hydroxide) by-products. Over time, the amorphous C-S-H gel deposited on the cement grows and hardens, thus gaining more strength as the setting proceeds, while portlandite released from cement increases the alkalinity of the environment. The C-S-H phase has a porous structure with negative surface charges due to which nucleation of calcium phosphate on the cement surface and formation of apatite-like materials occur [31]. The reaction of tricalcium aluminate and ferrite produces needle-form crystals of ettringite (hexacalcium aluminate trisulphate hydrate) and monosulphate phase [32–34]. SEM of hydrated Portland cement shows similar features to MTA including unhydrated cement grains, calcium hydroxide crystals (portlandite), and ettringite needles in addition to capillary cracks and voids [35].

The water/powder ratio has an influence on the rheological properties of the prepared cement and might affect the hydration rate and by-products [36]. The literature has suggested the water/powder ratio of 0.3 to 0.6 in order to obtain acceptable consistency that increases as the hydration progresses. The main initial and final setting times of Portland cement have been reported to be 180 and 240 minutes, respectively [37].

### 3.2. Pore Structure of Hydrated Cement

Since the durability and various characteristics including mechanical properties, permeability, shrinkage, corrosion resistance, and bioactivity of cement are related to its pore structure, a brief review on this issue would be worthwhile [38]. Pore size distribution and the influence of various factors such as water : powder ratio, environment pH, and curing time on this property have been widely studied. Using mercury intrusion porosimetry, Chen et al. claimed that porosity of Portland cement increases with the increasing water/powder ratio and decreases with the progressing hydration process during 28 days [39]. Long-term curing of Portland cement leads to further hydration and a decrease in permeability regardless of the water/powder ratio [40]. It is noteworthy that the pore volume of MTA is significantly less than that of white Portland cement [41]. Increasing hydration temperature also affects the microstructure of the cement and consequently decreases its mechanical properties by producing capillary pores. In other words, higher hydration temperature enhances the initial strength but lowers the strength of cement at later ages [42]. Overall, the hydration process is the basis for the modification of cement properties.

### 3.3. Modification of Setting Time

In the presence of additional synthetic C-S-H seeds, hydration of tricalcium silicate accelerates. Additional C-S-H seeds provide excessive nuclei available for growth and eliminate the induction period at the beginning of the hydration process. The mechanical and chemical properties of hydrated Portland cement depend heavily on the C/S ratio of the C-S-H phase, which can be manipulated by altering the structure of C-S-H seeds based on the field of application [43]. Previous studies have used glucose [44], calcium gluconate [45], calcium lactate gluconate [46], lactic acid [47], sodium sulfate [48], calcium nitrite/nitrate, calcium carbonate nanoparticles, calcium formate, and calcium chloride for regulating the Portland cement setting process [49–51]. Following the incorporation of different accelerators, pH, temperature, and various properties of cement are affected in multiple ways; thus, further assessments of mechanical and biological properties are required [52]. One of the most effective additives to reduce the setting time of Portland cement and MTA is calcium chloride, which alters the kinetics of tricalcium silicate hydration [53].

Carbonates such as sodium carbonate and calcium carbonate are another group of mostly used accelerators, which act as nucleating agents [54]. Sodium carbonate and sodium bicarbonate decrease the initial and final setting times of Portland cement by enhancing the formation of ettringite and can improve the early age compressive strength of material during the first 7 days, but they can deteriorate it at later ages by replacing the calcium and sodium ions and consequently disintegrating the C-S-H gel structure [50, 55–58]. Nitrite- and nitrate-based accelerators have shown similar behavior. Despite boosting early strength of cement, it seems that they decrease the C-S-H phase and portlandite, which results in reduced compressive strength after 3 days [59].

Further modifications such as including 30 wt% wollastonite (CaSiO_3_) can reduce the setting time to approximately 10 minutes [60]. Up to 10% incorporation of nanosilica to radiopaque Portland cement reduces its initial and final setting time without compromising the compressive and flexural strength [61]. Addition of 2-10% sodium-lithium and potassium-silicates accelerate the setting process [62], although converse results were reported regarding the addition of up to 15% sodium fluorosilicate [63].

Incorporation of titanium oxide into PC also accelerates early cement hydration and C-S-H gel production, decreases its water permeability, and improves durability. On the other hand, titanium oxide delays the late hydration process by blocking water access to unhydrated cement parts [64]. Of note, addition of 1% titanium oxide improves cement's compressive and flexural strength [65]. Sugar with a dosage of 0.05%-0.1% can act as a set accelerator for Portland cement type *Ι* without harming its mechanical properties, while fewer concentrations of sugar might retard the tricalcium silicate hydration [66].

Exclusion of gypsum in the last step of manufacturing results in a reduction of setting time without changing cement's other properties [67, 68]. The accelerated PC has shown favorable compressive strength and alkaline pH [69]. Another modification of Portland cement to achieve a more functional cement compared to MTA is removal of gypsum at the last stage of PC manufacturing and addition of 1.8%-2.4% polycarboxylate superplasticizer. This compound showed more flowability, which is worthwhile for clinical use [70].

Another way to alter the setting time of PC is by adjusting its particle size distribution [71]. Although finer cement requires less setting time, it might result in the production of greater porosity and increased risk of cracking and shrinkage due to the higher initial heat release and hydration rate in early times [72].

Various radiopacifiers including predominantly bismuth oxide and zirconium oxide are not incorporated into hydration product phases but exert a significant impact on the setting time of Portland cement, which is discussed below.

In conclusion, hydration process is the basis for the improvement of cement properties and determines the setting time, early strength, durability, and ultimately mechanical properties (compressive, bond, tensile, and flexural strength). Nevertheless, divergent results can be found concerning the effect of nanoreinforcements on the hydration of Portland cement, which necessitate applying other evaluation techniques.

## 4. Physicomechanical Properties

### 4.1. Compressive Strength

As explained above, compressive strength of Portland cement increases over time [73]. A comparison of strength values of Portland cement and MTA indicated no difference 7 days after mixing [28]. Islam et al. reported similar results during 3 days, although after 28 days, ordinary and white Portland cement showed less strength compared to MTA [5]. Camilleri corroborated these findings [74]. Conversely, Ber et al. reported higher compressive strength for MTA compared to Portland cement in 24 hours and noticeable less strength after 3 weeks. This is explainable by more bismuth oxide in MTA structure used by Ber et al. [75]. PC containing bismuth oxide has similar initial and final compressive strength to MTA [76]. Ultimately, it is worth mentioning that physical properties are strongly related to curing conditions [77].

### 4.2. Flexural Strength

There are limited studies on the flexural strength of PC in comparison with other endodontic cements [78, 79]. Contamination with blood or saliva has no significant effect on the flexural strength of Portland cement [80]. Resin-based pit and fissure sealant containing hydrated PC fillers did not exhibit acceptable flexural strength, and the amount of filler adversely affected this property [81].

### 4.3. Push-Out Bond Strength

In 2 mm from the root apex, resinous cements showed superior push-out bond strength compared to calcium silicate-based cements, and MTA had similar push-out bond strength to Portland cement containing calcium tungstate or zirconium oxide [82]. Nevertheless, higher values of push-out bond strength have been reported for Portland cement with bismuth oxide [76].

Biomineralization seems to enhance this property and the resistance against displacement of cement [83–85]. Some modifications such as addition of calcium chloride to PC results in improved push-out strength, while calcium hydroxide affects it adversely [76].

### 4.4. Bond Strength

Bond strength of Portland cement and MTA to resin cements has been evaluated after immersion in water. According to this study, bond strength of Portland cement was significantly low compared to that of glass ionomer and MTA [86].

### 4.5. Fracture Resistance

Portland cement does not seem to influence the fracture resistance of dentin during 12 weeks, while MTA improved this property [87]. Notwithstanding, due to the limited studies in this regard, the impact of Portland cement on fracture resistance of dentin remains unclear.

### 4.6. Sealing Ability

Sealing ability is defined as the potentiality of the material to prevent microleakage, a vital prerequisite for endodontic cements. Previously, various dental materials including calcium hydroxide, amalgam, restorative materials, and glass ionomer cements have been used to repair furcation or root perforations and stripping; however, MTA and Portland cement play the leading role in this field. On the whole, hydraulic cements have demonstrated good performance in sealing root and furcation perforations in comparison with other materials [88]*. In vitro* studies have evaluated the ability of Portland cement to prevent leakage by bacterial infiltration [89], dye leakage [90, 91], fluid infiltration [82, 92], bovine serum albumin leakage, and scanning electron microscopy [93, 94], and most of them claimed that Portland cement and MTA have similar sealing ability. A possible reason for the sealing ability of Portland cement is the slight expansion upon the setting [95]. Using methods described by *International Organization for Standardization (ISO*) 6876:2001, the average expansion has been reported to be 0.47% for white Portland cement and 0.45% for grey Portland cement, which were significantly more than that of MTA [5]. In this method, the dimensional change was measured only in one direction and the device showed a lack of sensitivity (±1 *μ*m).

### 4.7. Solubility

Over time, Portland cement may be washed out in aqueous environments. Usually, the cement industry faces wet conditions such as underwater concrete placement, which may affect the material's properties, not dissimilar to the conditions occurring during periapical surgery. Although there are conflicting results regarding the solubility degree of Portland cement, most studies agree that its level of solubility is in accordance with the American National Standard Institute/American Dental Association (ANSI/ADA) recommendations [96–98].

Some studies conducted based on the weight loss of cements in dried and hydrated forms reported greater solubility for Portland cement in comparison to MTA [5, 99, 100]. On the contrary, solubility of Portland cement has been reported to be less than that of MTA with less than 3% weight loss during 24 hours [101–103]. In agreement with these studies, immersion of Portland cement in Hank's balanced salt solution (HBSS) showed that Portland cement had less than 1% solubility during 28 days [95]. Long-term evaluation of Portland cement solubility in comparison with MTA also resulted in similar findings according to which MTA Angelus and MTA Bio had more solubility [104].

These inconsistent results might be attributed to the various investigated Portland cement and MTA types and more importantly the water : powder ratio [96]. Furthermore, it has been reported that the mixing method exerts a significant effect on the solubility of Portland cement as it increases by using an amalgamator and ultrasonic mixing techniques [105]. In contrast, Duque et al. claimed that mixing tricalcium silicate-based cements with ultrasonic technique reduces their solubility due to the homogenous distribution of small particles [106].

To overcome these application problems in concrete industries, an antiwashout admixture (i.e., methylcellulose) is added to the cement to increase the viscosity and cohesion of cement [107].

## 5. Biological Properties

### 5.1. Osteo/Odontogenic Potential of Portland Cement

Pulp repair during the pulpotomy or pulp capping depends on the ability of pulp capping material to induce pulp regeneration, mineralization, and hard tissue barrier formation because of odontoblast activation. Furthermore, osteogenesis has been observed after placing Portland cement and MTA implants in intraosseous sites of animals, confirming the osteoconductive behavior of these materials [108]. Studies have also evaluated biomineralization following the implantation of dentin tubes filled with Portland cement in the subcutaneous tissue of rats [109, 110]. Portland cement induces the expression of potent markers related to bone remodeling such as cytokines (IL-18, IL-1*β*, and IL-6) and osteocalcin in human osteosarcoma cells [111]. Furthermore, it allows odontoblastic differentiation of hDPCs and mineralization-related gene expression (osteonectin, alkaline phosphatase, dentin sialophosphoprotein, osteopontin, bone sialoprotein, and matrix extracellular phosphoglycoprotein) and consequently induces dentinogenesis [112–114]. Maher et al. reported enhanced cell proliferation and osteogenic differentiation of dental pulp pluripotent-like stem cells in a media pretreated with commercially available pure Portland cement [115].

Portland cement has a great ability to induce the mineral density in carious dentin which shows the remineralization potential of this material [116]. Previous studies have demonstrated the growth of crystalline deposits (carbonated apatite) in the dentin Portland cement interface in direct contact with phosphate-buffered saline [83, 85, 117, 118]. Expression of dental matrix protein-1 in teeth that received pulpotomy treatment with Portland cement confirms hard tissue barrier formation since DMP-1 is associated with differentiation of odontoblast-like and odontoblast cells and mineralization of dentin [119]. Portland cement also enhances differentiation and mineralization of the periodontal ligament cells by inducing expression of alkaline phosphatase, bone morphogenetic protein, and DMP-1 [120]. Histological analysis of pulp tissue of human primary teeth treated with Portland cement and MTA showed mineralized material deposition 6 months following pulp capping while approximately half of teeth treated with MTA exhibited formation of dentin bridge at this duration. Disappointedly, the calcium hydroxide group showed discrete necrotic areas and chronic inflammatory infiltrate [121].

### 5.2. Inflammatory Reaction to Portland Cement

Assessment of pulp condition after application of Portland cement for pulp capping has exhibited pulp repair with no inflammation and internal resorption [119]. In contrast, calcium hydroxide, formocresol, and zinc oxide eugenol have shown variable degrees of inflammatory infiltrates [119, 122].

Saidon et al. evaluated the tissue reaction of pig mandible bone to Portland cement after 12 weeks of implantation. Although a slight inflammatory response was detected, no chronic inflammatory cell was observed [108]. Histological analysis of dorsal connective tissue of rats following implantation of dentin tubes filled with Portland cement demonstrated moderate inflammatory response by mainly lymphocytes and macrophages during the first week, which decreased significantly after 60 days and was attributed to the alkaline pH and calcium release during the setting process. Furthermore, high expression of osteopontin was detected in the fibroblasts of surrounding tissue of implanted material [109]. A similar inflammatory response subsequent to Portland cement implantation at rat subcutaneous tissue was also observed in other studies [122–125]. According to Marques et al., no significant difference was between MTA Fillapex and Portland cement in the degree of inflammatory reaction; however, Portland cement showed more satisfactory results in tissue repair [122]. In a parallel study, Shahi et al. reported a severe infiltration following implantation of white and gray Portland cement and white and gray MTA during the first 7 days. Although gray Portland cement showed a significant decrease in inflammatory cells in the 60-day interval, there was a slight tendency to increase in the 90-day interval. The same trend was observed in the white Portland cement group between 30 and 60 days after implantation. In this study, gray MTA demonstrated more favorable biocompatibility [126].

### 5.3. Cytotoxicity of MGPC towards Various Cell Lines

Histological and immunochemistry analysis of human pulp tissue following pulpotomy and pulp capping using Portland cement-based materials has shown the reparative capacity and bioinductivity of these materials in addition to biocompatibility, nontoxicity, and nongenotoxicity [11, 13, 83]. Portland cement exhibits more biocompatibility in comparison with glass ionomer, calcium hydroxide, and zinc oxide [111, 112]. Numerous in vitro studies suggest the low cytotoxicity of Portland cement towards various cell lines and tissues including human endothelial cells [127], rat osteosarcoma cells [128], human osteosarcoma cells [68, 111], human bone marrow-derived mesenchymal stem cells [129], human peripheral lymphocytes [11], human osteoblasts [130], mouse lymphoma cells [131], and Chinese hamster ovary cells [13]. More importantly, the biological response of human dental pulp cells (hDPCs) and human periodontal ligament fibroblasts (hPLFs) to Portland cement has been analyzed extensively, and no significant cytotoxicity has been reported [113, 114, 132–134]. As evidenced by SEM, hDPSCs cultured on Portland cement were flat and had well-formed cytoplasmic extensions branching off from cells to adjacent cells and areas [111–113]. Portland cement and MTA have demonstrated similar effects on the growth pattern of hDPSCs, although D' Anto et al. reported that MTA supports tissue regeneration through enhancement of human mesenchymal stem cell (hMSC) adhesion, proliferation, and migration better than Portland cement [129]. In an investigation on hPLFs comparing Portland cement, MT,A and amalgam, Portland cement and MTA showed greater results in expression of essential extracellular matrix proteins including collagen type *Ι* and fibronectin as well as TGF-*β* [134].

## 6. Antimicrobial Activity

Portland cement's high alkalinity is a proposed mechanism for its antimicrobial activity. Portland cement's pH rises from 7 to 12.3 upon hydration and continues to increase for 3 hours to reach a pH of 12.9. It is noteworthy that since the cement manufacturing process requires a temperature of 15000°C, the commercial samples are generally sterile. Contaminated samples might be suitably sterilized using dry heat sterilization or autoclaving; the physical properties require further evaluation after the process [111]. Studies assessing the antibacterial efficacy of Portland cement by the agar diffusion method have reported equivocal results. In a study on various endodontic cements against *Enterococcus faecalis*, *Staphylococcus aureus*, *Pseudomonas aeruginosa*, *Bacillus subtilis*, and *Candida albicans*, Portland cement and MTA showed diffusion in agar, although no inhibitory effect was observed. Calcium hydroxide exhibited the best antimicrobial activity [135]. MTA and Portland cement have not shown significant antimicrobial effects compared to calcium hydroxide against *E. faecalis*, *P. aeruginosa*, *S. aureus*, *Escherichia coli*, and *Bacteroides fragilis* [136, 137]. In agreement with these studies, Miyagak et al. reported no antibacterial and antifungal efficacy using MTA, Portland cement, and Sealapex while AH Plus showed a considerable inhibitory effect. *E. faecalis* was resistant to all t5ested sealers [138]. Comparing the antibacterial activity of pure Portland cement and Portland cement impregnated with silver nanoparticles shows no inhibitory effect of pure Portland cement against *Streptococcus mutans* [139]. In contrast, Tanomaru-Filho et al. found that white and gray Portland cement exhibited a similar inhibition zone against *E. faecalis*, *P. aeruginosa*, *C. albicans*, *S. aureus*, and *E. coli* species. However, the antimicrobial activity of Sealapex with zinc oxide, zinc oxide eugenol, and Sealer 26 was significantly higher [10]. In a parallel study, Portland cement, MTA, Sealapex, and Fill Canal showed acceptable antimicrobial activity except against *E. coli* [140].

### 6.1. Antimicrobial Activity of MGPC Containing a Radiopacifier

It has been shown that white Portland cement, MTA Angelus, and radiopaque Portland cement containing zirconium oxide or niobium oxide possess similar remarkable antimicrobial activity against *E. faecalis*, *P. aeruginosa*, *C. albicans*, *S. mutans*, and *Kocuria rhizophila* [141]. In another study, addition of radiopacifiers including bismuth carbonate, bismuth subnitrate, and zirconium oxide to Portland cement did not seem to increase antibacterial activity, while a remarkable improvement in antifungal activity was observed [142]. The incorporation of nanozirconium oxide into Portland cement did not affect the antimicrobial activity against *S. aureus* and *E. coli*, while it increased this activity against *P. aeruginosa* [143]. Furthermore, addition of nanohydroxyapatite and silver nanoparticles to MTA and radiopaque Portland cement increased the antibacterial activity of cements in both planktonic and biofilm forms [144, 145]. These conflicting results regarding the antimicrobial activity of root canal filling materials might be attributed to various investigated microorganisms, different compositions of the sealers on the market, cement concentration, and application of freshly mixed or set sealer [146].

## 7. Drawbacks

### 7.1. Heavy Metal Leaching

According to the consensus regarding the necessity of the absence of heavy metal leaching from endodontic materials, clinical applications of Portland cement are a cause for concern. High concentrations of chromium, arsenic, and lead released from Portland cement in physiological solutions have been reported; thus, the biocompatibility and safety of Portland cement are still ambiguous [15, 147]. Conversely, some researchers reported lower levels of arsenic release below the limit considered to be harmful [148, 149]. Using high-performance liquid chromatography (HPLC), De-Deus et al. reported no significant difference between the arsenic release of various on the market MTA and Portland cement [149]. Evaluation of the heavy metal leaching behavior from white and gray Portland cement and ProRoot MTA and MTA Angelus by atomic absorption spectrophotometry showed that gray Portland cement releases the most chromium and arsenic amounts [147, 150]. Furthermore, lead was detected only in gray Portland cement. Another study by Bramante et al. reported that only white Portland cement and white MTA release arsenic below the limit recommended by ISO 9917-1 and [151]. In conclusion, clinical applications of gray Portland cement might proceed with caution.

### 7.2. Tooth Color Alteration

The grayish color of Portland cement is due to the elements such as magnesium and iron. Amongst the Portland cement-based materials, Portland cement has been reported to exhibit the best color stability, although it was not significantly different from white MTA after 12 months [152]. The significant discoloration was detected when bismuth oxide was added to Portland cement while Portland cement with zirconium oxide presents acceptable color stability [153]. In contrast, it has been reported that teeth in contact with Portland cement containing 20% zirconium oxide or calcium tungstate and MTA Angelus present significant color alterations after 30 days. Although there is no significant difference between the Δ*E* of mentioned materials, Portland cement containing zirconium oxide had more luminosity compared to MTA [154]. Color stability of Portland cement exposed to light and anaerobic conditions or formalin and hydroxyethylmethacrylate (HEMA) solutions also has been shown [155, 156]. Discoloration of MTA and bismuth oxide containing Portland cement is associated with the interaction of bismuth oxide and dentin collagen. Furthermore, decomposition of bismuth oxide at high temperatures results in the formation of oxygen and bismuth which is responsible for darkening the material [157, 158]. Overall, most studies agree on the color stability of Portland cement with zirconium oxide as a radiopacifier compared to various MTA types.

## 8. Conclusion

This review outlines the evolving knowledge regarding the various properties of Portland cement and provides a deeper insight into the behavior of this material during its lifetime. Although mainly articles published in dental journals are discussed, some studies on Portland cement concrete are included. Heavy metal leaching and some concerns over the mechanical properties of Portland cement restrain its clinical application; however, histological analysis has shown favorable results in terms of biocompatibility, differentiation, and proliferation of hDPCs with a negligible inflammatory reaction of the pulpal tissue. Based on the current studies, Portland cement is a bioactive endodontic cement that could be considered an alternative to MTA providing further investigations. Developed materials from Portland cement predominantly show the same characteristics and even some of them surpass the qualities of the base material. However, while Portland cement was applied as the base material of grey MTAs, many years ago, it is not permitted to be used as a medical device in recent years. An endodontic medical grade cement should be produced over strictly controlled processes to be considered as a dentistry device. It is vital to further address the applicability, toxicity, and the other properties of PC before transferring into day-to-day clinical practice.

## References

[1] Parirokh M., Torabinejad M. (2010). Mineral Trioxide Aggregate: A Comprehensive Literature Review--Part III: Clinical Applications, Drawbacks, and Mechanism of Action. *Journal of Endodontics*.

[2] Ishikawa K., Ducheyne P. (2017). 1.17 bioactive ceramics: cements. *Comprehensive Biomaterials II*.

[3] Singh N. (2020). Properties of cement and concrete in presence of nanomaterials. *Smart Nanoconcretes and Cement-Based Materials*.

[4] Chattopadhyay B. (2020). Genetically-enriched microbe-facilitated self-healing nano-concrete. *Smart Nanoconcretes and Cement-Based Materials*.

[5] Islam I., Kheng Chng H., Jin Yap A. U. (2006). Comparison of the physical and mechanical properties of MTA and Portland cement. *Journal of Endodontics*.

[6] Parker G. (2001). *Encyclopedia of materials: science and technology*.

[7] Dunstetter F., De Noirfontaine M.-N., Courtial M. (2006). Polymorphism of tricalcium silicate, the major compound of Portland cement clinker: 1. Structural data: review and unified analysis. *Cement and Concrete Research*.

[8] Cuesta A., Ayuela A., Aranda M. A. (2021). Belite cements and their activation. *Cement and Concrete Research*.

[9] Neville A. M. (1995). *Properties of concrete*.

[10] Tanomaru-Filho M., Tanomaru J. M., Barros D. B., Watanabe E., Ito I. Y. (2007). In vitro antimicrobial activity of endodontic sealers, MTA-based cements and Portland cement. *Journal of Oral Science*.

[11] Braz M. G., Camargo E., Salvadori D. M. F., Marques M., Ribeiro D. (2006). Evaluation of genetic damage in human peripheral lymphocytes exposed to mineral trioxide aggregate and Portland cements. *Journal of Oral Rehabilitation*.

[12] Juárez Broon N., Bramante C. M., Assis G. F. (2006). Healing of root perforations treated with mineral trioxide aggregate (MTA) and Portland cement. *Journal of Applied Oral Science*.

[13] Ribeiro D. A., Sugui M. M., Matsumoto M. A., Duarte M. A. H., Marques M. E. A., Salvadori D. M. F. (2006). Genotoxicity and cytotoxicity of mineral trioxide aggregate and regular and white Portland cements on Chinese hamster ovary (CHO) cells in vitro. *Oral Surgery, Oral Medicine, Oral Pathology, Oral Radiology, and Endodontology*.

[14] Torabinejad M., Chivian N. (1999). Clinical applications of mineral trioxide aggregate. *Journal of Endodontics*.

[15] Camilleri J., Kralj P., Veber M., Sinagra E. (2012). Characterization and analyses of acid-extractable and leached trace elements in dental cements. *International Endodontic Journal*.

[16] Dammaschke T., Gerth H. U., Züchner H., Schäfer E. (2005). Chemical and physical surface and bulk material characterization of white ProRoot MTA and two Portland cements. *Dental Materials*.

[17] Belío-Reyes I. A., Bucio L., Cruz-Chavez E. (2009). Phase Composition of ProRoot Mineral Trioxide Aggregate by X-Ray Powder Diffraction. *Journal of Endodontics*.

[18] Cianconi L., Palopoli P., Campanella V., Mancini M. (2016). Composition and microstructure of MTA and Aureoseal Plus: XRF, EDS, XRD and FESEM evaluation. *European Journal of Paediatric Dentistry*.

[19] Asgary S., Parirokh M., Eghbal M. J., Brink F. (2004). A comparative study of white mineral trioxide aggregate and white Portland cements using X-ray microanalysis. *Australian Endodontic Journal*.

[20] Funteas U. R., Wallace J., Fochtman F. (2003). A comparative analysis of mineral trioxide aggregate and Portland cement. *Australian Endodontic Journal*.

[21] de Oliveira M. G., Xavier C. B., Demarco F. F., Pinheiro A. L. B., Costa A. T., Pozza D. H. (2007). Comparative chemical study of MTA and Portland cements. *Brazilian Dental Journal*.

[22] Camilleri J. (2008). Characterization and chemical activity of Portland cement and two experimental cements with potential for use in dentistry. *International Endodontic Journal*.

[23] Khalil I., Naaman A., Camilleri J. (2015). Investigation of a novel mechanically mixed mineral trioxide aggregate (MM-MTA(™)). *International Endodontic Journal*.

[24] Song J.-S., Mante F. K., Romanow W. J., Kim S. (2006). Chemical analysis of powder and set forms of Portland cement, gray ProRoot MTA, white ProRoot MTA, and gray MTA-Angelus. *Oral Surgery, Oral Medicine, Oral Pathology, Oral Radiology, and Endodontology*.

[25] Hwang Y. C., Lee S. H., Hwang I. N. (2009). Chemical composition, radiopacity, and biocompatibility of Portland cement with bismuth oxide. *Oral Surgery, Oral Medicine, Oral Pathology, Oral Radiology, and Endodontology*.

[26] Camilleri J., Montesin F. E., Curtis R. V., Ford T. R. P. (2006). Characterization of Portland cement for use as a dental restorative material. *Dental Materials*.

[27] Bozeman T. B., Lemon R. R., Eleazer P. D. (2006). Elemental analysis of crystal precipitate from gray and white MTA. *Journal of Eendodontics*.

[28] Hwang Y.-C., Kim D.-H., Hwang I.-N. (2011). Chemical constitution, physical properties, and biocompatibility of experimentally manufactured Portland cement. *Journal of Endodontics*.

[29] Ha W. N., Shakibaie F., Kahler B., Walsh L. J. (2016). Deconvolution of the particle size distribution of ProRoot MTA and MTA Angelus. *Acta Biomaterialia Odontologica Scandinavica*.

[30] Komabayashi T., Spångberg L. S. (2008). Comparative analysis of the particle size and shape of commercially available mineral trioxide aggregates and Portland cement: a study with a flow particle image analyzer. *Journal of Endodontics*.

[31] Prati C., Gandolfi M. G. (2015). Calcium silicate bioactive cements: biological perspectives and clinical applications. *Dental Materials*.

[32] Gandolfi M. G., Van Landuyt K., Taddei P., Modena E., Van Meerbeek B., Prati C. (2010). Environmental scanning electron microscopy connected with energy dispersive x-ray analysis and Raman techniques to study ProRoot mineral trioxide aggregate and calcium silicate cements in wet conditions and in real time. *Journal of Endodontics*.

[33] Camilleri J. (2007). Hydration mechanisms of mineral trioxide aggregate. *International Endodontic Journal*.

[34] Camilleri J., Sorrentino F., Damidot D. (2013). Investigation of the hydration and bioactivity of radiopacified tricalcium silicate cement, biodentine and MTA Angelus. *Dental Materials*.

[35] Camilleri J., Cutajar A., Mallia B. (2011). Hydration characteristics of zirconium oxide replaced Portland cement for use as a root-end filling material. *Dental Materials*.

[36] Röbler M., Odler I. (1985). Investigations on the relationship between porosity, structure and strength of hydrated Portland cement pastes I. Effect of porosity. *Cement and Concrete Research*.

[37] Ylmén R., Jäglid U., Steenari B.-M., Panas I. (2009). Early hydration and setting of Portland cement monitored by IR, SEM and Vicat techniques. *Cement and Concrete Research*.

[38] Odler I., Rößler M. (1985). Investigations on the relationship between porosity, structure and strength of hydrated Portland cement pastes. II. Effect of pore structure and of degree of hydration. *Cement and Concrete Research*.

[39] Chen X., Wu S., Zhou J. (2014). Experimental study and analytical model for pore structure of hydrated cement paste. *Applied Clay Science*.

[40] Tracz T., Zdeb T. (2019). Effect of hydration and carbonation progress on the porosity and permeability of cement pastes. *Materials*.

[41] Saghiri M. A., Asgar K., Lotfi M., Karamifar K., Neelakantan P., Ricci J. L. (2012). Application of mercury intrusion porosimetry for studying the porosity of mineral trioxide aggregate at two different pH. *Acta Odontologica Scandinavica*.

[42] Bahafid S., Ghabezloo S., Faure P., Duc M., Sulem J. (2018). Effect of the hydration temperature on the pore structure of cement paste: experimental investigation and micromechanical modelling. *Cement and Concrete Research*.

[43] Thomas J. J., Jennings H. M., Chen J. J. (2009). Influence of nucleation seeding on the hydration mechanisms of tricalcium silicate and cement. *The Journal of Physical Chemistry C*.

[44] Tenoutasse N. (1978). Effect of glucose and calcium gluconate on the hydration of Portland cement. *Indian Journal of Technology*.

[45] Singh N. (1976). Effect of gluconates on the hydration of cement. *Cement and Concrete Research*.

[46] Hsieh S.-C., Teng N.-C., Lin Y.-C. (2009). A novel accelerator for improving the handling properties of dental filling materials. *Journal of Endodontics*.

[47] Singh N., Prabha S., Singh A. (1986). Effect of lactic acid on the hydration of Portland cement. *Cement and Concrete Research*.

[48] Lee T., Lee J., Choi H. (2020). Effects of accelerators and retarders in early strength development of concrete based on low-temperature-cured ordinary Portland and calcium sulfoaluminate cement blends. *Materials*.

[49] Machado D. F. M., Bertassoni L. E., Souza E. M. ., Almeida J. B. ., Rached R. N. (2010). Effect of additives on the compressive strength and setting time of a Portland cement. *Brazilian Oral Research*.

[50] Teixeira C. D. S., Wasielewsky J. C., Santos G. S., Bernardi A., Bortoluzzi E. A., Garcia L. . F. R. (2021). Effect of the addition of nanoparticles ofCaCO3and different water-to-powder ratios on the physicochemical properties of white Portland cement. *Microscopy Research and Technique*.

[51] Wiltbank K. B., Schwartz S. A., Schindler W. G. (2007). Effect of selected accelerants on the physical properties of mineral trioxide aggregate and Portland cement. *Journal of Endodontics*.

[52] Bodanezi A., Carvalho N., Silva D. (2008). Immediate and delayed solubility of mineral trioxide aggregate and Portland cement. *Journal of Applied Oral Science*.

[53] Bortoluzzi E. A., Broon N. J., Bramante C. M., Felippe W. T., Tanomaru Filho M., Esberard R. M. (2009). The influence of calcium chloride on the setting time, solubility, disintegration, and pH of mineral trioxide aggregate and white Portland cement with a radiopacifier. *Journal of Endodontics*.

[54] Bernardi A., Bortoluzzi E. A., Felippe W. T., Felippe M. C., Wan W. S., Teixeira C. S. (2017). Effects of the addition of nanoparticulate calcium carbonate on setting time, dimensional change, compressive strength, solubility and pH of MTA. *International Endodontic Journal*.

[55] Wang Y., He F., Wang J., Hu Q. (2019). Comparison of effects of sodium bicarbonate and sodium carbonate on the hydration and properties of Portland cement paste. *Materials*.

[56] Camiletti J., Soliman A. M., Nehdi M. L. (2013). Effect of nano-calcium carbonate on early-age properties of ultra-high-performance concrete. *Magazine of Concrete Research*.

[57] Cao M., Ming X., He K., Li L., Shen S. (2019). Effect of macro-, micro- and nano-calcium carbonate on properties of cementitious composites—a review. *Materials*.

[58] Supit S. W. M., Shaikh F. U. A. (2014). Effect of Nano-CaCO<sub>3</sub> on compressive strength development of high volume fly ash mortars and concretes. *Journal of Advanced Concrete Technology*.

[59] Choi H., Inoue M., Choi H. (2019). Physicochemical study on the strength development characteristics of cold weather concrete using a nitrite-nitrate based accelerator. *Materials*.

[60] Flores-Ledesma A., Barceló Santana F., Bucio L., Arenas-Alatorre J., Faraji M., Wintergerst A. (2017). Bioactive materials improve some physical properties of a MTA-like cement. *Materials Science and Engineering: C*.

[61] Akbari M., Zebarjad S. M., Nategh B., Rouhani A. (2013). Effect of nano silica on setting time and physical properties of mineral trioxide aggregate. *Journal of Endodontics*.

[62] Witzleben S. T. (2020). Acceleration of Portland cement with lithium, sodium and potassium silicates and hydroxides. *Materials Chemistry and Physics*.

[63] Appelbaum K. S., Stewart J. T., Hartwell G. R. (2012). Effect of sodium fluorosilicate on the properties of Portland cement. *Journal of Endodontics*.

[64] Diamantopoulos G., Katsiotis M., Fardis M. (2020). The role of titanium dioxide on the hydration of portland cement: a combined NMR and ultrasonic study. *Molecules*.

[65] Khataee R., Heydari V., Moradkhannejhad L., Safarpour M., Joo S. W. (2013). Self-cleaning and mechanical properties of modified white cement with nanostructured TiO2. *Journal of Nanoscience and Nanotechnology*.

[66] Ahmad S., Lawan A., Al-Osta M. (2020). Effect of sugar dosage on setting time, microstructure and strength of type I and type V Portland cements. *Case Studies in Construction Materials*.

[67] Camilleri J., Montesin F. E., Brady K., Sweeney R., Curtis R. V., Ford T. R. P. (2005). The constitution of mineral trioxide aggregate. *Dental Materials*.

[68] Camilleri J., Montesin F., di Silvio L., Pitt Ford T. (2005). The chemical constitution and biocompatibility of accelerated Portland cement for endodontic use. *International Endodontic Journal*.

[69] Camilleri J. (2008). The physical properties of accelerated Portland cement for endodontic use. *International Endodontic Journal*.

[70] Wongkornchaowalit N., Lertchirakarn V. (2011). Setting time and flowability of accelerated Portland cement mixed with polycarboxylate superplasticizer. *Journal of Endodontics*.

[71] Ha W. N., Bentz D. P., Kahler B., Walsh L. J. (2015). D90: the strongest contributor to setting time in mineral trioxide aggregate and Portland cement. *Journal of Endodontics*.

[72] Bentz D. P., Garboczi E. J., Haecker C. J., Jensen O. M. (1999). Effects of cement particle size distribution on performance properties of Portland cement-based materials. *Cement and Concrete Research*.

[73] Bortoluzzi E. A., Cassel de Araújo T., Carolina Corrêa Néis A. (2019). Effect of different water-to-powder ratios on the dimensional stability and compressive strength of mineral aggregate-based cements. *European Oral Research*.

[74] Camilleri J. (2010). Evaluation of the physical properties of an endodontic Portland cement incorporating alternative radiopacifiers used as root-end filling material. *International Endodontic Journal*.

[75] Ber B. S., Hatton J. F., Stewart G. P. (2007). Chemical modification of ProRoot MTA to improve handling characteristics and decrease setting time. *Journal of Endodontics*.

[76] Negm A., Hassanien E., Abu-Seida A., Nagy M. (2016). Physical evaluation of a new pulp capping material developed from Portland cement. *Journal of Clinical and Experimental Dentistry*.

[77] Formosa L. M., Mallia B., Camilleri J. (2012). The effect of curing conditions on the physical properties of tricalcium silicate cement for use as a dental biomaterial. *International Endodontic Journal*.

[78] Wu Y. Y., Que L., Cui Z., Lambert P. (2019). Physical properties of concrete containing graphene oxide nanosheets. *Materials*.

[79] Jee H., Park J., Zalnezhad E. (2019). Characterization of titanium nanotube reinforced cementitious composites: mechanical properties, microstructure, and hydration. *Materials*.

[80] Alhodiry W., Lyons M. F., Chadwick R. G. (2014). Effect of saliva and blood contamination on the bi-axial flexural strength and setting time of two calcium-silicate based cements: Portland cement and biodentine. *European Journal of Prosthodontics*.

[81] Yang S. Y., Choi J. W., Kim K. M., Kwon J. S. (2020). Prevention of secondary caries using resin-based pit and fissure sealants containing hydrated calcium silicate. *Polymers*.

[82] Amoroso-Silva P. A., Marciano M. A., Guimarães B. M., Duarte M. A. H., Sanson A. F., Moraes I. G. (2014). Apical adaptation, sealing ability and push-out bond strength of five root-end filling materials. *Brazilian Oral Research*.

[83] Reyes-Carmona J. F., Felippe M. S., Felippe W. T. (2009). Biomineralization ability and interaction of mineral trioxide aggregate and white Portland cement with dentin in a phosphate-containing fluid. *Journal of Endodontics*.

[84] Iacono F., Gandolfi M. G., Huffman B. (2010). Push-out strength of modified Portland cements and resins. *American Journal of Dentistry*.

[85] Reyes-Carmona J. F., Felippe M. S., Felippe W. T. (2010). The biomineralization ability of mineral trioxide aggregate and Portland cement on dentin enhances the push-out strength. *Journal of Endodontics*.

[86] Lemos Martins Sicuro S., Gabardo M. C., Castiglia Gonzaga C. (2016). Bond strength of self-adhesive resin cement to different root perforation materials. *Journal of Endodontics*.

[87] Forghani M., Bidar M., Shahrami F., Bagheri M., Mohammadi M., Attaran Mashhadi N. (2013). Effect of MTA and Portland cement on fracture resistance of dentin. *Journal of Dental Research Dental Clinics Dental Prospects*.

[88] Aqrabawi J. (2000). Sealing ability of amalgam, super EBA cement, and MTA when used as retrograde filling materials. *British Dental Journal*.

[89] De-Deus G., Petruccelli V., Gurgel-Filho E., Coutinho-Filho T. (2006). MTA versus Portland cement as repair material for furcal perforations: a laboratory study using a polymicrobial leakage model. *International Endodontic Journal*.

[90] Coneglian P. Z. A., Orosco F. A., Bramante C. M., Moraes I. G. ., Garcia R. B., Bernardineli N. (2007). In vitro sealing ability of white and gray mineral trioxide aggregate (MTA) and white Portland cement used as apical plugs. *Journal of Applied Oral Science*.

[91] El Tawil S. B., El Dokkyl N., El Hamid D. (2011). Sealing ability of MTA versus Portland cement in the repair of furcal perforations of primary molars: a dye extraction leakage model. *Journal of American Science*.

[92] De-Deus G., Reis C., Brandão C., Fidel S., Fidel R. A. S. (2007). The Ability of Portland Cement, MTA, and MTA Bio to Prevent Through-and- Through Fluid Movement in Repaired Furcal Perforations. *Journal of Endodontics*.

[93] Chittoni S. B., Martini T., Wagner M. H. (2012). Back-scattered electron imaging for leakage analysis of four retrofilling materials. *Microscopy Research and Technique*.

[94] Baranwal A. K., Paul M. L., Mazumdar D., Adhikari H. D., Vyavahare N. K., Jhajharia K. (2015). An ex-vivo comparative study of root-end marginal adaptation using grey mineral trioxide aggregate, white mineral trioxide aggregate, and Portland cement under scanning electron microscopy. *Journal of Conservative Dentistry: JCD*.

[95] Camilleri J. (2011). Evaluation of the effect of intrinsic material properties and ambient conditions on the dimensional stability of white mineral trioxide aggregate and Portland cement. *Journal of Endodontics*.

[96] Parirokh M., Torabinejad M. (2010). Mineral Trioxide Aggregate: A Comprehensive Literature Review--Part I: Chemical, Physical, and Antibacterial Properties. *Journal of Endodontics*.

[97] Dorileo M. C. G. O., Villa R. D., Guedes O. A. (2014). Comparative analysis of selected physicochemical properties of Pozzolan Portland and MTA-based cements. *International Scholarly Research Notices*.

[98] Borges Á. H., Pedro F. L., Miranda C. E., Semenoff-Segundo A., Pécora J. D., Filho A. M. C. (2010). Comparative study of physico-chemical properties of MTA-based and Portland cements. *Acta Odontológica Latinoamericana*.

[99] Danesh G., Dammaschke T., Gerth H., Zandbiglari T., Schafer E. (2006). A comparative study of selected properties of ProRoot mineral trioxide aggregate and two Portland cements. *International Endodontic Journal*.

[100] Dorileo M. C., Pedro F. L., Bandeca M. C., Guedes O. A., Villa R. D., Borges A. H. (2014). Comparative analysis of physicochemical properties of root perforation sealer materials. *Restorative Dentistry and Endodontics*.

[101] Kim J., Kim H., Chang S. W., Kim S., Choi K. K., Jang J. H. (2021). Effect of bioactive glass addition on the physical properties of mineral trioxide aggregate. *Biomaterials Research*.

[102] Hungaro Duarte M. A., Minotti P. G., Rodrigues C. T. (2012). Effect of different radiopacifying agents on the physicochemical properties of white Portland cement and white mineral trioxide aggregate. *Journal of Endodontics*.

[103] de Souza L. C., Yadlapati M., Lopes H. P., Silva R., Letra A., Elias C. N. (2017). Physico-chemical and biological properties of a new Portland cement-based root repair material. *Eurasian Endodontics Journal*.

[104] Vivan R. R., Zapata R. O., Zeferino M. A. (2010). Evaluation of the physical and chemical properties of two commercial and three experimental root-end filling materials. *Oral Surgery, Oral Medicine, Oral Pathology, Oral Radiology, and Endodontology*.

[105] Shahi S., Ghasemi N., Rahimi S., Yavari H., Samiei M., Jafari F. (2016). Effect of different mixing methods on the physical properties of Portland cement. *Journal of Clinical and Experimental Dentistry*.

[106] Duque J. A., Fernandes S. L., Bubola J., Duarte M. A. H., Camilleri J., Marciano M. A. (2018). The effect of mixing method on tricalcium silicate-based cement. *International Endodontic Journal*.

[107] Khayat K. H. (1998). Viscosity-enhancing admixtures for cement-based materials -- An overview. *Cement and Concrete Composites*.

[108] Saidon J., He J., Zhu Q., Safavi K., Spångberg L. S. (2003). Cell and tissue reactions to mineral trioxide aggregate and Portland cement. *Oral Surgery, Oral Medicine, Oral Pathology, Oral Radiology, and Endodontology*.

[109] Viana Viola N., Maria Guerreiro-Tanomaru J., Ferreira da Silva G., Sasso-Cerri E., Tanomaru-Filho M., Cerri P. S. (2012). Biocompatibility of an experimental MTA sealer implanted in the rat subcutaneous: quantitative and immunohistochemical evaluation. *Journal of Biomedical Materials Research Part B: Applied Biomaterials*.

[110] Dreger L. A. S., Felippe W. T., Reyes-Carmona J. F., Felippe G. S., Bortoluzzi E. A., Felippe M. C. S. (2012). Mineral Trioxide Aggregate and Portland Cement Promote Biomineralization _In Vivo_. *Journal of Endodontics*.

[111] Abdullah D., Pitt Ford T. R., Papaioannou S., Nicholson J., McDonald F. (2002). An evaluation of accelerated Portland cement as a restorative material. *Biomaterials*.

[112] Min K.-S., Kim H.-I., Park H.-J., Pi S.-H., Hong C.-U., Kim E.-C. (2007). Human pulp cells response to Portland cement in vitro. *Journal of Endodontics*.

[113] Min K.-S., Lee S.-I., Lee Y., Kim E.-C. (2009). Effect of radiopaque Portland cement on mineralization in human dental pulp cells. *Oral Surgery, Oral Medicine, Oral Pathology, Oral Radiology, and Endodontology*.

[114] Lee S.-K., Lee S.-K., Lee S.-I. (2010). Effect of calcium phosphate cements on growth and odontoblastic differentiation in human dental pulp cells. *Journal of Endodontics*.

[115] Maher A., Núñez-Toldrà R., Carrio N. (2018). The effect of commercially available endodontic cements and biomaterials on osteogenic differentiation of dental pulp pluripotent-like stem cells. *Dentistry Journal*.

[116] Neves A. B., Bergstrom T. G., Fonseca-Gonçalves A., Dos Santos T. M. P., Lopes R. T., de Almeida Neves A. (2019). Mineral density changes in bovine carious dentin after treatment with bioactive dental cements: a comparative micro-CT study. *Clinical Oral Investigations*.

[117] Tay F. R., Pashley D. H., Rueggeberg F. A., Loushine R. J., Weller R. N. (2007). Calcium phosphate phase transformation produced by the interaction of the Portland cement component of white mineral trioxide aggregate with a phosphate-containing fluid. *Journal of Endodontics*.

[118] Meschi N., Li X., Van Gorp G., Camilleri J., Van Meerbeek B., Lambrechts P. (2019). Bioactivity potential of Portland cement in regenerative endodontic procedures: from clinic to lab. *Dental Materials*.

[119] Lourenço Neto N., Marques N. C., Fernandes A. P. (2016). Immunolocalization of dentin matrix protein-1 in human primary teeth treated with different pulp capping materials. *Journal of Biomedical Materials Research Part B: Applied Biomaterials*.

[120] Wang M.-C., Yeh L.-Y., Shih W.-Y., Li W.-C., Chang K.-W., Lin S.-C. (2018). Portland cement induces human periodontal ligament cells to differentiate by upregulating miR-146a. *Journal of the Formosan Medical Association*.

[121] Oliveira T., Moretti A., Sakai V. T. (2013). Clinical, radiographic and histologic analysis of the effects of pulp capping materials used in pulpotomies of human primary teeth. *European Archives of Paediatric Dentistry*.

[122] Marques N. C. T., Lourenço Neto N., Fernandes A. P., Rodini C. O., Duarte M. A. H., Oliveira T. M. (2013). Rat subcutaneous tissue response to MTA Fillapex® and Portland cement. *Brazilian Dental Journal*.

[123] Lourenço Neto N., Marques N. C. T., Paula Fernandes A. (2014). Biocompatibility of Portland cement combined with different radiopacifying agents. *Journal of Oral Science*.

[124] Mangala M., Sharath Chandra S. M., Bhavle R. M. (2015). To evaluate the biocompatibility of the Indian Portland cement with potential for use in dentistry: an animal study. *Journal of Conservative Dentistry: JCD*.

[125] Koçak S., Erten H., Baris E., Türk S., Alaçam T. (2014). Evaluation of the biocompatibility of experimentally manufactured Portland cement: an animal study. *Journal of Clinical and Experimental Dentistry*.

[126] Shahi S., Rahimi S., Yavari H. R. (2010). Effect of mineral trioxide aggregates and Portland cements on inflammatory cells. *Journal of Endodontics*.

[127] de Deus G., Ximenes R., Gurgel-Filho E., Plotkowski M., Coutinho-Filho T. (2005). Cytotoxicity of MTA and Portland cement on human ECV 304 endothelial cells. *International Endodontic Journal*.

[128] Gomes Cornélio A. L., Salles L. P., Campos da Paz M., Cirelli J. A., Guerreiro-Tanomaru J. M., Tanomaru Filho M. (2011). Cytotoxicity of Portland cement with different radiopacifying agents: a cell death study. *Journal of Endodontics*.

[129] D'Antò V., Di Caprio M. P., Ametrano G., Simeone M., Rengo S., Spagnuolo G. (2010). Effect of mineral trioxide aggregate on mesenchymal stem cells. *Journal of Endodontics*.

[130] Bidar M., Tavakkol Afshari J., Shahrami F. (2007). Evaluation of adhesion and morphology of human osteoblasts to white MTA and Portland cement. *Iranian Endodontic Journal*.

[131] Ribeiro D. A., Duarte M. A. H., Matsumoto M. A., Marques M. E. A., Salvadori D. M. F. (2005). Biocompatibility in vitro tests of mineral trioxide aggregate and regular and white Portland cements. *Journal of Endodontics*.

[132] Yoshino P., Nishiyama C. K., Modena K. C. . S., Santos C. F., Sipert C. R. (2013). In vitro cytotoxicity of white MTA, MTA Fillapex® and Portland cement on human periodontal ligament fibroblasts. *Brazilian Dental Journal*.

[133] Cai S., Zhang W., Tribble G., Chen W. (2017). Reactions of human dental pulp cells to capping agents in the presence or absence of bacterial exposure. *Journal of Oral Science*.

[134] Fayazi S., Razmi H., Ostad S. N. (2011). Effect of ProRoot MTA, Portland cement, and amalgam on the expression of fibronectin, collagen I, and TGF*β* by human periodontal ligament fibroblasts in vitro. *Indian Journal of Dental Research*.

[135] Estrela C., Bammann L. L., Estrela C. R., Silva R. S., Pecora J. D. (2000). *Antimicrobial and chemical study of MTA, Portland cement, calcium hydroxide paste, Sealapex and Dycal*.

[136] Asgary S., Kamrani F. A. (2008). Antibacterial effects of five different root canal sealing materials. *Journal of Oral Science*.

[137] Ribeiro C. S., Kuteken F. A., Hirata Júnior R., Scelza M. F. Z. (2006). Comparative evaluation of antimicrobial action of MTA, calcium hydroxide and Portland cement. *Journal of Applied Oral Science*.

[138] Miyagak D. C., Carvalho E. M. O. F. ., Robazza C. R. C., Chavasco J. K., Levorato G. L. (2006). In vitro evaluation of the antimicrobial activity of endodontic sealers. *Brazilian Oral Research*.

[139] Nam K. Y. (2017). Characterization and antimicrobial efficacy of Portland cement impregnated with silver nanoparticles. *The Journal of Advanced Prosthodontics*.

[140] Sipert C., Hussne R., Nishiyama C., Torres S. (2005). In vitro antimicrobial activity of Fill Canal, Sealapex, mineral trioxide aggregate, Portland cement and EndoRez. *International Endodontic Journal*.

[141] Tanomaru J. M. G., Storto I., Da Silva G. F. (2014). Radiopacity, pH and antimicrobial activity of Portland cement associated with micro- and nanoparticles of zirconium oxide and niobium oxide. *Dental Materials Journal*.

[142] Weckwerth P. H., Machado A. C. . O., Kuga M. C., Vivan R. R., Polleto R. . S., Duarte M. A. H. (2012). Influence of radiopacifying agents on the solubility, pH and antimicrobial activity of Portland cement. *Brazilian Dental Journal*.

[143] Li Q., Coleman N. J. (2014). Hydration kinetics, ion-release and antimicrobial properties of white Portland cement blended with zirconium oxide nanoparticles. *Dental Materials Journal*.

[144] Vazquez-Garcia F., Tanomaru-Filho M., Chávez-Andrade G. M. (2016). Effect of silver nanoparticles on physicochemical and antibacterial properties of calcium silicate cements. *Brazilian Dental Journal*.

[145] Guerreiro-Tanomaru J. M., Vazquez-Garcia F. A., Bosso-Martelo R., Bernardi M. I. B., Faria G., Tanomaru Filho M. (2016). Effect of addition of nano-hydroxyapatite on physico-chemical and antibiofilm properties of calcium silicate cements. *Journal of Applied Oral Science*.

[146] Eldeniz A. U., Hadimli H. H., Ataoglu H., Ørstavik D. (2006). Antibacterial effect of selected root-end filling materials. *Journal of Endodontics*.

[147] Schembri M., Peplow G., Camilleri J. (2010). Analyses of heavy metals in mineral trioxide aggregate and Portland cement. *Journal of Endodontics*.

[148] Duarte M. A. H., de Oliveira Demarchi A. C. C., Yamashita J. C., Kuga M. C., de Campos Fraga S. (2005). Arsenic release provided by MTA and Portland cement. *Oral Surgery, Oral Medicine, Oral Pathology, Oral Radiology, and Endodontology*.

[149] de-Deus G., de Souza M. C. B., Sergio Fidel R. A., Fidel S. R., de Campos R. C., Luna A. S. (2009). Negligible expression of arsenic in some commercially available brands of Portland cement and mineral trioxide aggregate. *Journal of Endodontics*.

[150] Chang S. W., Shon W. J., Lee W., Kum K. Y., Baek S. H., Bae K. S. (2010). Analysis of heavy metal contents in gray and white MTA and 2 kinds of Portland cement: a preliminary study. *Oral Surgery, Oral Medicine, Oral Pathology, Oral Radiology, and Endodontology*.

[151] Monteiro Bramante C., Demarchi A. C. C. O., de Moraes I. G. (2008). Presence of arsenic in different types of MTA and white and gray Portland cement. *Oral Surgery, Oral Medicine, Oral Pathology, Oral Radiology, and Endodontology*.

[152] Lenherr P., Allgayer N., Weiger R., Filippi A., Attin T., Krastl G. (2012). Tooth discoloration induced by endodontic materials: a laboratory study. *International Endodontic Journal*.

[153] Dettwiler C. A., Walter M., Zaugg L. K., Lenherr P., Weiger R., Krastl G. (2016). In vitro assessment of the tooth staining potential of endodontic materials in a bovine tooth model. *Dental Traumatology*.

[154] Marciano M. A., Costa R. M., Camilleri J., Mondelli R. F., Guimarães B. M., Duarte M. A. (2014). Assessment of color stability of white mineral trioxide aggregate angelus and bismuth oxide in contact with tooth structure. *Journal of Endodontics*.

[155] Vallés M., Mercadé M., Duran-Sindreu F., Bourdelande J. L., Roig M. (2013). Influence of Light and Oxygen on the Color Stability of Five Calcium Silicate- based Materials. *Journal of Endodontics*.

[156] Berger T., Baratz A. Z., Gutmann J. L. (2014). In vitro investigations into the etiology of mineral trioxide tooth staining. *Journal of Conservative Dentistry: JCD*.

[157] Sanz O., Haro-Poniatowski E., Gonzalo J., Fernández Navarro J. M. (2006). Influence of the melting conditions of heavy metal oxide glasses containing bismuth oxide on their optical absorption. *Journal of Non-Crystalline Solids*.

[158] Marciano M. A., Duarte M. A. H., Camilleri J. (2015). Dental discoloration caused by bismuth oxide in MTA in the presence of sodium hypochlorite. *Clinical Oral Investigations*.

